# Mechanistic and Structural Studies of Protein-Only RNase P Compared to Ribonucleoproteins Reveal the Two Faces of the Same Enzymatic Activity

**DOI:** 10.3390/biom6030030

**Published:** 2016-06-24

**Authors:** Cédric Schelcher, Claude Sauter, Philippe Giegé

**Affiliations:** 1 UPR 2357, Centre National de la Recherche Scientifique, Institut de Biologie Moléculaire des Plantes, Université de Strasbourg, 12 rue du général Zimmer, F-67084 Strasbourg, France; cedric.schelcher@etu.unistra.fr; 2 UPR 9002, Centre National de la Recherche Scientifique, Architecture et Réactivité de l’ARN, Institut de Biologie Moléculaire et Cellulaire, Université de Strasbourg, 15 rue René Descartes, Strasbourg F-67084, France; c.sauter@unistra.fr

**Keywords:** tRNA biogenesis, RNase P, PRORP, crystal structures, kinetic analyses

## Abstract

RNase P, the essential activity that performs the 5′ maturation of tRNA precursors, can be achieved either by ribonucleoproteins containing a ribozyme present in the three domains of life or by protein-only enzymes called protein-only RNase P (PRORP) that occur in eukaryote nuclei and organelles. A fast growing list of studies has investigated three-dimensional structures and mode of action of PRORP proteins. Results suggest that similar to ribozymes, PRORP proteins have two main domains. A clear functional analogy can be drawn between the specificity domain of the RNase P ribozyme and PRORP pentatricopeptide repeat domain, and between the ribozyme catalytic domain and PRORP N4BP1, YacP-like Nuclease domain. Moreover, both types of enzymes appear to dock with the acceptor arm of tRNA precursors and make specific contacts with the corner of pre-tRNAs. While some clear differences can still be delineated between PRORP and ribonucleoprotein (RNP) RNase P, the two types of enzymes seem to use, fundamentally, the same catalytic mechanism involving two metal ions. The occurrence of PRORP and RNP RNase P represents a remarkable example of convergent evolution. It might be the unique witness of an ongoing replacement of catalytic RNAs by proteins for enzymatic activities.

## 1. Introduction

Similar to Janus, the two-faced Roman god of transitions and passages that concomitantly looks to the future and to the past, RNase P has two faces. This essential enzymatic activity that removes the 5′ leader sequences of tRNA precursors occurs either as ribonucleoproteins (RNP) involving a ribozyme [1] or as protein-only enzymes [2,3,4,5]. The ancestral RNP RNase P, first described in *Escherichia coli* [6,7,8] is ubiquitous in bacteria and archaea, although it is absent from an archaea species where tRNA transcription starts at position +1 and RNase P is not needed [9]. RNP RNase P also occurs in the nucleus and/or organelles of a variety of eukaryotes, notably in animal nuclei and in both mitochondria and the nucleus in fungi [10]. Nevertheless, a completely distinct form of RNase P devoid of RNA also appeared during eukaryote evolution and is present in the nucleus and/or organelles of several eukaryotes [11].

RNP RNases P are present in all three domains of life, their catalytic RNA (P RNA) structure is relatively well conserved in prokaryotes and eukaryote nuclei [10]. However, it is highly degenerate in several organelles [12]. P RNAs contain two main domains, i.e., a catalytic (C) and a specificity (S) domain. The protein content of RNP RNase P is comparatively more divergent. While a single protein subunit is found in bacteria, 4 to 5 subunits occur in archaea and up to 10 in eukaryote nuclei [10,13,14,15,16].

In contrast, protein-only RNase P enzymes are entirely specific to eukaryotes. These enzymes, termed PRORP (for PROtein-only RNase P or PROteinaceous RNase P), have two main domains; a C-terminal catalytic domain belonging to the N4BP1, YacP-like Nuclease (NYN) family [17] and an N-terminal pentatricopeptide repeat (PPR) domain involved in RNA binding [18]. The two main domains are linked by a connecting domain involving zinc [19,20]. PRORP proteins were initially described in human mitochondria and in the organelles and nucleus of the model plant *Arabidopsis thaliana* [20,21,22,23]. They were also characterized in *Trypanosoma brucei* nucleus and mitochondria [24], and in other species of the green lineage, i.e., *Ostreococcus tauri* [25], the moss *Physcomitrella patens* [26], and the model green algae *Chlamydomonas reinhardtii* [27]. Interestingly, while separate RNase P enzymes were always found in the nucleus and organelles of eukaryotes (either multiple RNPs, multiple PRORPs or a combination of both), *Chlamydomonas* utilizes a single PRORP protein for RNase P activity in the nucleus, mitochondria and the chloroplast, thus making the most compact and versatile RNase P machinery described to date in both prokaryotes and eukaryotes [27]. Likewise, PRORP sequences are also found in a diversity of other eukaryotes, in particular in Stramenopiles, Alveolata, and Rhizaria. PRORP thus occurs in four out of five main eukaryote super-groups, but never co-exists with RNPs in the same compartment. Indeed, the occurrence of PRORP and RNPs seems mutually exclusive in cell compartments where gene expression takes place, or even in entire organisms [11]. For instance, although RNase P RNA and PRORP have both been proposed to be present in human mitochondria [28], PRORP appears to be sole responsible for RNase P in this compartment [21,29]. Remarkably, PRORP proteins are capable of functionally replacing RNPs for RNase P activity, as shown by complementation experiments in *Escherichia coli* [22] and *Saccharomyces cerevisiae* [30], thus showing that the two types of enzymes are fully interchangeable, at least for the maturation of essential substrates, such as pre-tRNAs. In some cases, eukaryote RNPs might have been retained because they evolved additional functions that could not be performed by PRORP, e.g., as observed in human nuclei with the requirement of RNP RNase P for the formation of RNA polymerase III initiation complexes [31]. Nonetheless, RNase P activity makes a unique example of an activity that can be performed either by RNA or by proteins. It might be the witness of a still ongoing transition from the ancestral RNA world, where molecular processes were catalyzed by RNA molecules, to the contemporary world, where enzymatic activities are primarily held by proteins.

Here, we review the biochemical, structural, and mechanistic data obtained for protein-only RNase P and discuss how PRORP structure, pre-tRNA recognition, and cleavage differ, but also resemble that of RNP RNase P.

## 2. Diversity of PRORP Sequence Features

Functionally characterized, as well as putative, PRORP sequences are all typified by an α-superhelical domain containing tandem arrays of PPR motifs and a specific NYN metallonuclease domain, bridged by a bipartite zinc-binding domain. Sequences of the N-terminal PPR domains are highly degenerate, but recognizable, by the 3D fold specific for PPR motifs [11,18]. Recent advances on PPR proteins’ mode of action suggest that each PPR motif specifically binds a single nucleotide in RNA substrates, according to a “PPR code” where the nature of residues, in particular at positions five and 35 of the respective motifs, is responsible for the specificity of the motif for a given nucleotide [32,33,34,35]. A longer stretch of PPR motifs would, thus, bind a higher number of nucleotides. Among PRORP sequences, the numbers of PPR motifs predicted with TPRpred [11] range from two to four. In addition, structure predictions systematically reveal variable numbers of additional PPR-like motifs [11]. This variability in the size and nature of PPR domains suggests that the respective PRORP enzymes might have different RNA binding properties, e.g., variable affinities for RNA substrates.

The C-terminal regions of PRORP enzymes contain a metallonuclease domain that was shown to be responsible for the endonuclease activity [22]. This domain belongs to the PIN-like super-family of nucleases and, more precisely, to the NYN family. The actual NYN domain of PRORP appears to be of prokaryote origin as it resembles putative bacterial YacP ribonuclease sequences. *YacP* genes are ubiquitous in prokaryotes and eukaryotes, with the notable exception of fungi [17]. While NYN domain proteins are widespread in eukaryotes, the NYN domains of PRORP proteins have specificities that allow discrimination between bona fide PRORP proteins and other NYN domain proteins. They are characterized by a specific signature involving conserved aspartates and histidines, i.e., composed by a first motif (D/E/T/H/N/P/G)h_3_D(G/A)xN and a second motif DDx_15–39_(S/T)xDx_3_DH separated by 50 to 80 amino acids. This NYN signature of PRORP differs significantly from the basic NYN signature [17], and also differs to some degree from the Pfam RNase_Zc3h12a signature (PF11977). Although conserved in PRORP, the NYN domains have lineage-specific disparities, e.g., the spacer sequence between the two motifs is typically longer in plants as compared to animals [11].

The connecting zinc-binding domain is split into two subparts upstream and downstream of the NYN domain. Its first part involves a highly conserved CxxC motif, where both cysteines were shown to be required for zinc coordination in Arabidopsis and human [19,20,36]. However, the CxxC motif is not ubiquitous in PRORP. For instance, in the Chlorophyta *Chlamydomonas* CxxC is replaced by CxxA [27]. The second part of the zinc-binding domain is defined by (W/Y/F)HxPx and (W/F)xCx_2-3_(R/K) signatures [11]. Here, as well, lineage-specific variations are observed. Altogether, this variability in the connecting domain suggests that the respective PRORP enzymes might bind zinc and/or other metals with different affinities.

Beyond the PPR, the NYN and the connecting domain, other signatures are present in PRORP sequences of specific phyla. For example, a glycine-rich insertion is characteristic of land plant PRORPs. In some phyla, a PPP(Y/F/C)(S/T) motif is found between the NYN domain and the second zinc-binding subdomain. The occurrence of such specific insertions might indicate additional phylum-specific functions, e.g., through interactions with yet unidentified proteins.

## 3. Comparison of PRORP Three-Dimensional Structures

First analyses of PRORP sequences along with biochemical and biophysical studies, including synchrotron radiation circular dichroism and small angle X-ray scattering, showed that PRORP enzymes are organized in two main domains and contain a high proportion of α-helices [20,22]. This was confirmed by the determination of atomic resolution structures of Arabidopsis organellar PRORP1 and nuclear PRORP2 as well as of human PRORP (previously referred to as mitochondrial ribonuclease P protein 3, MRPP3) by X-ray crystallography [19,36,37,38,39]. These structures reveal a largely similar architecture and, thus, highlight the overall conservation of the PRORP fold in distantly related eukaryotic phyla.

### 3.1. Common Structural Features

**Figure 1:**
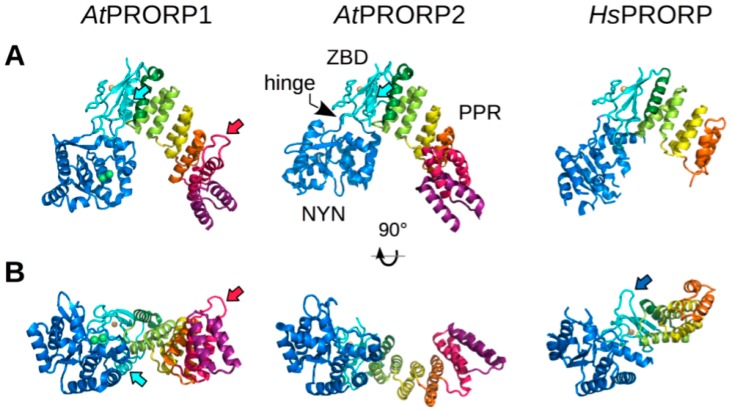
Protein-only RNase P (PRORP) architecture. As of May 2016 nine crystal structures of PRORP enzymes are available in the Protein Data Bank, four for Arabidopsis PRORP1 in the presence of different metal ions (PDB IDs: 4G24, 4G25, 4G26), two for PRORP2 (5DIZ, 5TF9) and three for truncated forms of human PRORP lacking PPR1-4 or PPR1-2 modules (4ROU, 4XGL, 4XGM). Selected structures correspond to *At*PRORP1 with two bound manganese ions shown in green (4G24, 1.95 Å resolution, [19]), and highest resolution models of *At*PRORP2 (5FT9, 3.05 Å resolution, Pinker et al. in preparation) and of *Hs*PRORP (4XGL, 1.8 Å resolution, [36]). All PRORP enzymes contain an N-terminal pentatricopeptide repeat (PPR) domain made of five PPR modules (PPR 1-5 depicted in violet, red, orange, yellow, and green, respectively) followed by a single helix (half PPR or PPR5B, shown in dark green), and a C-terminal N4BP1, YacP-like Nuclease (NYN) catalytic domain (NYN, depicted in dark blue). These two functional domains are linked by a bipartite zinc binding domain (ZBD, depicted in cyan) coordinating a Zn^2+^ ion (shown in gold). PRORP structures are either superimposed according to their PPR3-5B modules and ZBD (**A**) or to their catalytic domain (**B**). These two orthogonal views highlight the possible reorientation of PPR and NYN domains with respect to each other, owing to a flexible hinge present between the NYN and ZBD. Red, cyan, and blue arrows indicate the long loop of PPR2 motif present in *At*PRORP1, the 30-residue-long linker inserted in the ZBD next to the PPR domain in Arabidopsis enzymes, and the extended lariat loop in the central region of human PRORP, respectively. Molecular representations of PRORPs were prepared with PyMOL (Schrödinger, Portland, OR, USA).

As expected, the N-terminal PPR domain forms a superhelical structure closely resembling that of TPR (tetratricopeptide repeat) motifs, a domain evolutionary related to PPR and involved in protein-protein interactions [40,41]. Despite the high primary sequence divergence between Arabidopsis and human PRORP sequences, their PPR motifs are, structurally, highly similar (Figure 1). They are also superimposable with other motifs from PPR proteins of established 3D structure [42,43,44,45], thus emphasizing the strong structural conservation of PPR motifs among eukaryotes.

Then, the catalytic domain of PRORP implements an α/β/α sandwich fold typical of all structurally-characterized PIN-like and flap nucleases [17,46,47]. A comparable fold is also present in the ribonuclease MCP-1 induced protein 1 (MCPIP1) that takes part in the regulation of the immune response through the degradation of inflammatory cytokines mRNAs [47]. Similarly, the nuclease domains of T4 RNase H [48] and of SMG6 and SMG5, two essential proteins involved in nonsense-mediated mRNA decay in humans, also adopt a similar fold [46]. The crystal structure of PRORP1 revealed that four aspartate residues are involved in the binding of metal ions [19] contrary to flap nucleases that utilize six conserved aspartates [17]. Among the PRORP NYN domain, four positions (D399, D474, D475, and D493 in Arabidopsis PRORP1) are strictly conserved in all sequences and were shown to be essential for pre-tRNA maturation [20]. In other proteins containing a NYN domain and not involved in RNase P activity, the positions equivalent to D399, D475, and D493 are also conserved, but position 474 is often an alanine [17].

The two main domains are bridged by the zinc-binding domain, which appears to have a structural role, i.e., to connect the two functional domains and, thus, to give PRORP its overall structural design (Figure 1). The zinc-binding domain forms the apex of PRORP characteristic Λ shape. In this configuration, the PPR and NYN domains form the two arms of the Λ. On one side, the concave part of the PPR superhelix, that provides a platform for interaction with RNA if PRORP conforms to the overall mode of action of PPR proteins [43,49], faces the catalytic groove on the other side of the Λ, thus exposing conserved aspartate residues and metal ions toward the predicted RNA binding side of the protein.

### 3.2. Specific Structural Features

The comparison of Arabidopsis organellar PRORP1 with nuclear PRORP2 structures revealed that PPR domains in both enzymes are very similar and composed of five and a half PPR and PPR-like motifs corresponding to 11 consecutive α-helices. However, differences are found: for instance, motif PPR2 of PRORP1 has an extension between its two α-helices (Figure 1). The function of this loop is unknown, but might be involved in protein-protein interactions that specifically take place in Arabidopsis organelles. While PRORP1 and 2 share the same overall Λ-shaped structure, the angle of the Λ differs significantly (Figure 1A), the angle in PRORP2 being more open than that in PRORP1 [19,39]. This difference could reflect specificities of organellar and nuclear PRORP modes of action. This is, however, unlikely because both PRORP1 and 2 appear to be able to cleave any pre-tRNA of canonical fold, as reviewed below, and pre-tRNA substrates have a canonical fold in both the Arabidopsis nucleus and organelles (contrary to animals, where mitochondrial tRNA structures often differ significantly from nuclear encoded tRNA structures [50]). A more likely hypothesis is that the two crystal structures have captured two alternate conformations that can be adopted by PRORP proteins. PRORP1 structure shows structural domains organized in a plane (Figure 1B), whereas PRORP2 and human PRORP structures display a left or right rotation of PPR with respect to NYN domains. This twist is allowed by the presence of a hinge between the catalytic and zinc-binding domains [36,39]. Human PRORP, as PRORP2, adopts a more relaxed Λ shape. Such conformational flexibility/adaptability, which can be detected in solution by small-angle X-ray scattering (SAXS) (Pinker et al., in preparation) might be required for PRORP function, e.g., to select and bind precursor substrates, and release products.

Similarly, the comparison of human PRORP structure with plant PRORP structures revealed some discrepancies. Plant PRORP proteins feature a 30-residue-long loop-helix-loop linker inserted between the PPR and the zinc-binding domains which is replaced by a short loop in the human enzyme. In turn the latter has a longer lariat loop in its zinc-binding connecting domain as compared to Arabidopsis PRORPs. The human structure is stabilized by an extended hydrogen bond network and makes a finger between the PPR and NYN domain (Figure 1B), whereas it adopts a more compact horseshoe-like bend in plants. The longer lariat in humans does not seem to be involved in binding TRMT10C and SDR5C1, the two additional subunits of human mitochondrial RNase P [21,51], as its exchange with a shorter linker did not result in enzyme activity loss in the presence of the two partners. The loop substitution did not make PRORP work independently from its partners either [36]. This discrepancy might, alternatively, be related to intrinsic differences in PRORP potential for flexibility or structural changes in plants and animals. Still, the reason why human PRORP requires two protein cofactors is not clear yet. One hypothesis is that the NYN domain contains structural features, like the lariat loop, that inhibit its activity in the absence of partners. PRORP chimers, in which the whole human NYN domain was substituted by that of PRORP1, were used in an attempt to create a cofactor-independent PRORP [36,37], but only the latter group succeeded in partially restoring an activity. It was also proposed that the alternate conformation triggered by a salt bridge with a neighboring arginine in the loop containing the two conserved aspartates could hamper binding of one metal ion required for catalysis [37]. However, the absence of metal ions in the catalytic site is not specific to human PRORP. Two (independent) crystallographic studies of PRORP2 led to the same observation of a metal-free NYN domain. Overall, these data are rather in favor of a role of TRMT10C and SDR5C1 as structural chaperones to select and stabilize an active conformation of human PRORP. The fact that only mutants with truncated PPR domains could be crystallized, not a full-length construct, suggests that PRORP is, indeed, a dynamic protein.

## 4. Mechanistic Analyses of Protein-Only RNase P Activity

The capacity of PRORP enzymes to functionally replace RNP enzymes in vivo suggested that both types of RNase P might share a similar mode of action. For its activity, bacterial RNP RNase P docks onto the acceptor arm of the pre-tRNA substrate. In particular, the P RNA specificity (S) domain interacts with the tRNA corner formed by the stacked T and D loops and the catalytic (C) domain contacts the cleavage site between nucleotides −1 and +1 of pre-tRNAs [52,53,54]. Then, the pre-tRNA leader sequences interact with the protein subunit of the RNP [55]. Finally, in *E. coli*, the 3′ terminal CCA of tRNAs binds a complementary sequence in a P RNA loop of RNP RNase P. The initial analysis of PRORP bipartite organization suggested that the PPR domain of PRORP might play a role similar to that of the P RNA S domain, while the NYN domain would have a function similar to that of the C domain in RNP RNase P [5]. A growing body of evidence now suggests that PRORP indeed conforms to that model, although its mode of action also differs in some aspects to that of RNP RNase P.

### 4.1. Kinetic Analyses of PRORP Activity

A number of studies have investigated the activity and kinetic constants of PRORP cleavage of pre-tRNAs. Activity assays were performed with the three Arabidopsis PRORP enzymes, with plant organellar and nuclear pre-tRNAs, as well as with bacterial pre-tRNA substrates. Reactions were performed as single or multiple turnover kinetics experiments. Results summarized in Table 1 reveal major kinetic parameter disparities between individual studies, depending on the protein used and/or the pre-tRNA investigated. For instance, k_obs_ vary from 1 to 7 min^−1^, K_D_ values determined with a same protein but different substrates range from 60 to 2300 nM, while K_M_ values determined in multiple turnover conditions vary from 140 to 2000 nM (Table 1) and can be as low as 3 nM with a bacterial substrate cleaved by PRORP3 in single turnover conditions [56] (Table 2). The detailed comparison of results suggests that the three Arabidopsis PRORP enzymes perform RNase P activity with comparable catalytic efficiencies. However, some pre-tRNA substrates appear to be cleaved more efficiently by a given PRORP paralogue. This might reflect true, although subtle, differences in the mode of action of organellar versus nuclear PRORP enzymes. The two types of PRORP enzymes might have slightly different substrate recognition processes [57] which might reflect the co-evolution of a given enzyme with its substrate population in vivo. In other cases, the observed kinetic parameters discrepancies most probably also reflect differences in experimental setups and the intrinsic nature of pre-tRNAs used in the respective studies. For instance, tRNAs with a highly stable acceptor stem, such as *Thermus thermophilus* pre-tRNA^Gly^, are most probably inherently better RNase P substrates than other pre-tRNAs with less stable structures, such as Arabidopsis mitochondrial pre-tRNA^Cys^.

### 4.2. Involvement of PRORP *cis*-Elements for RNase P Activity

**Figure 2:**
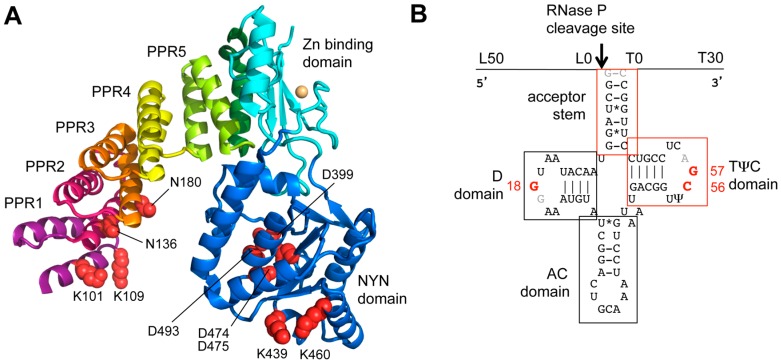
PRORP and pre-tRNA *cis*-elements required for RNase P activity. (**A**) Arabidopsis PRORP1 crystal structure shows the three domain organization of PRORP, with the PPR and NYN domains connected by a zinc-binding domain. Side chains of residues that were shown to interact with pre-tRNA and/or to be essential for RNase P activity by mutagenesis analyses (see Table 1) are shown in space-filling mode (red spheres); and (**B**) a secondary structure model of Arabidopsis mitochondrial pre-tRNA^Cys^. L and T indicate the leader and trailer sequences and their respective length spans in the different substrates used in the analyses summarized in Table 2. tRNA domains and residues that were found to be essential for RNase P activity are indicated in red. Other positions where mutations had no effect on activity are shown in grey.

Beyond the analysis of wild-type PRORP proteins activity, some studies have investigated the variations of PRORP activity and kinetic parameters in PRORP mutants. Results summarized in Table 1 confirm the essential role of the four conserved aspartates (D399, D474 D475, and D493 in Arabidopsis PRORP1) of the NYN domain for RNase P cleavage. Results also show that different deletions of N-terminal parts of PRORP, especially deletions of PPR motifs strongly affect RNase P activity. In the PPR domain of plant PRORPs, positions 5 and 35 of motif PPR3 are the most conserved and point mutations at these positions strongly affect RNase P activity [56,58]. However, in the latter studies, mutations in the PPR domain were analyzed by RNase P cleavage assays and direct RNA binding was not analyzed. Results thus represent indirect evidence to show the importance of the PPR domain for RNA recognition and do not conclusively show if and/or how PPR motifs confer specificity to PRORP enzymes. In another study, lysine residues positioned at both tips of PRORP Λ shape were found in contact with the pre-tRNA substrate [59], thus, in agreement with the general substrate binding process proposed for PRORP proteins [5]. Mutated residues that were shown to interact with pre-tRNA and/or to be essential for RNase P activity are shown in Figure 2.

### 4.3. Requirements of Pre-tRNA Substrate *cis*-elements for PRORP Activity

Similarly, a number of studies have investigated the nature of pre-tRNA *cis*-elements required for PRORP mediated RNase P cleavage. For this, activity assays were performed with collections of either plant or bacterial pre-tRNA substrates, with varying leader and trailer sequences lengths with domain deletions as well as point mutations. Reactions were performed with the three Arabidopsis PRORP paralogues during single or multiple turnover kinetics experiments. Results summarized in Table 2 suggest that contrary to bacterial RNP RNase P, the leader and trailer sequences are not involved in substrate binding by PRORP. Optimal cleavage efficiencies could be obtained with leader sequences of at least two nucleotides and no longer than 10 nucleotides (Table 2). Transcripts with longer leaders might form alternative secondary structures in vitro that prevent the binding of PRORP, thus decreasing the apparent cleavage efficiency. Similarly, contrary to bacteria, the nature of the residue immediately upstream of the pre-tRNA cleavage site does not seem to be of primary importance for PRORP activity. Catalysis takes place with the four possible residues, although its efficiency increased two-fold when an A was immediately upstream of the cleavage site [56].

Deletions of tRNA domains revealed their respective importance for PRORP activity. While the deletion of the anticodon domain of pre-tRNAs did not affect RNase P activity, deletions of the D and T domains dramatically affected RNase P activity. Still, highly reduced RNase P activity could be obtained with minimal substrates composed of the acceptor and T domains, suggesting that interaction with PRORP is somehow possible in the absence of the tRNA D domain [56]. However, other minimal substrates containing the acceptor and D domains only were not investigated, thus preventing to find out whether minimal activity could also take place in the absence of the tRNA T domain. Nonetheless, results suggest that both the D and especially the T domain of pre-tRNAs play a critical role in RNase P cleavage by PRORP.

Nucleotide substitutions in pre-tRNA substrates helped map more precisely the residues required for PRORP activity. Results suggest that individual residues are necessary for RNase P activity in both the D and T loop of pre-tRNAs, in particular, G18 in the D loop and both C56 and a purine at position 57 (Table 2). Moreover, footprint experiments showed protection by PRORP for residues belonging to the D and T loops of pre-tRNAs, thus confirming PRORP contact with the corner of pre-tRNAs [20]. Altogether, results suggest that PRORP, similar to bacterial RNP RNase P docks onto the acceptor arm of pre-tRNAs, with no contact with the anticodon arm and appears to specifically interact with residues from the D and T loop at the corner of tRNAs.

### 4.4. PRORP Cleavage Mechanism

Beyond the common architectural features and substrate specificities of PRORP and RNP RNase P for pre-tRNA binding, the two types of biocatalyst appear to share a similar catalytic mechanism [5,19,60]. The RNP enzyme was shown to use a two-metal-ion mechanism to cleave the phosphodiester bond [54,61]. It proceeds through a nucleophilic attack of the hydroxide ion apical to the O3′ of the upstream ribose, which generates products with 5′-phosphoryl and 3′-hydroxyl termini. The presence of two Mn^2+^ ions in the structure of Arabidopsis PRORP1 and metal dependency (Mn^2+^ or Mg^2+^) for PRORP cleavage suggest that protein-only enzymes also use a two-metal-ion mechanism to produce the attacking hydroxide ion and stabilize the transition state [19]. Nonetheless, PRORP1 is able to cleave substrates with an *R*_P_-phosphorothioate modification at the canonical cleavage site using Mg^2+^ as a cofactor, which is not the case of ribozyme RNase P [60,62]. This suggests that the *pro-R*_P_-oxygen of the scissile phosphate is not directly coordinated by a metal in PRORP active site, and suggests that the whole active site may adopt a different configuration. PRORP are proposed to use one hydrated divalent cation to provide the attacking hydroxide and a second hydrated metal ion to protonate the leaving group like the RNase P ribozyme [63,64].

In the latter study, Howard et al. also propose a model where the binding of one Mg^2+^ ion in the catalytic site increases the affinity of the second metal ion resulting in the cooperativity observed at low concentrations of pre-tRNAs. A comparison of the proposed mechanisms for PRORP and ribozyme RNase P activities shows that both enzymes require catalytic metal ions, although the metal ligands are different. Conserved aspartate residues coordinate the metal ions in PRORP, whereas ribozyme RNase P mainly uses non-bridging phosphodiester oxygens for metal ion coordination.

## 5. Comparison of PRORP and RNP RNase P Modes of Action

Altogether, biochemical studies published since the first descriptions of PRORP enzymes reveal that they share common features with RNP RNase P, but also diverge in many aspects. Alongside the discovery of protein-only RNase P, initial appreciations anticipated that the single subunit protein-only RNase P might be a much more efficient enzyme than RNP RNase P and that higher catalytic efficiency might have been the driving force for the replacement of ribozyme RNase P by PRORP during evolution. Pre-tRNA binding and cleavage studies of PRORP now suggest that it is not the case. For example, binding affinities of PRORP proteins for their pre-tRNA substrate are in the micromolar range [19,60]. These values are one or two orders of magnitude lower than for RNP RNases P. This might indicate that PRORP interaction with pre-tRNA substrates is more transient than that of RNP RNase P. Still, kinetic parameters of PRORP activity were monitored in vitro, and it cannot be ruled out that PRORP RNA binding affinity or catalytic efficiency might be higher in vivo, e.g., in a specific biochemical environment where the respective PRORP proteins accumulate and/or in complex with other factors that might modulate PRORP activity in vivo.

RNA binding assays revealed that the two types of enzymes are clearly distinct at the level of substrate recognition in the vicinity of the cleavage point. Hence, PRORP binding to pre-tRNA does not seem to involve specific interactions with pre-tRNA leader and trailer sequences contrary to RNP RNase P. In contrast, the two types of enzyme have a similar organization with the specificity and catalytic domains of P RNAs being analogous to the PPR and NYN domains of PRORP proteins. Moreover, the two types of enzymes appear to dock on the acceptor arm of pre-tRNAs and to make contact with specific residues at the corner of tRNAs formed by the stacking of T and D loops of tRNAs. However, despite these important similarities, PRORP cannot be considered as a true structural mimic of the RNase P ribozyme, at least not in the sense where the exact three-dimensional shape of a nucleic acid is mimicked by a protein for functional reasons, e.g., pathogen proteins shaped as DNA to avoid host defenses or ribosome recycling factors shaped as a tRNA to dissociate ribosomal subunits [65]. Similarities in substrate recognition processes of the two types of RNase P enzymes rather point to a remarkable case of convergent evolution, where a protein-only enzyme that appeared in eukaryotes independently evolved a substrate binding process reminiscent from that of the ancestral RNA-based RNase P.

## 6. Concluding Remarks

While major progress has been achieved to understand the biological functions [5] and evolution of protein-only RNase P [11], as well as to determine its three-dimensional architecture and mode of action (reviewed here), major questions remain. One of the key issues will be to precisely understand how substrate specificity is achieved and what the exact involvement of the individual PPR motifs in this process is. It was predicted that separate PPR motifs of PRORP might specifically interact with individual residues in the T and/or D loops of pre-tRNAs [5,56,58]. If PPR motifs of PRORP conform to the overall mode of action of PPR proteins [32,33,34,35] this would imply that PPR motifs of PRORP probably interact with the Watson-Crick side of residues, such as C56, R57, or G18. This would, in turn, imply that pre-tRNA substrates recognized by PRORP are not stabilized by Watson-Crick interactions that occur in mature tRNAs, i.e., the C56-G19 interaction or the G18-Ψ55 interaction. This would mean that these interactions that are required for the overall structure and stability of mature tRNAs [66] are only established upon release of the 5′ mature tRNA products from PRORP. The precise understanding of PRORP substrate specificity and the definition of its minimal substrate might give clues to identify transcriptome wide spectra of PRORP substrates in vivo. Similar to RNP RNase P that are involved in the maturation of a wide array of transcripts beyond pre-tRNAs [14], PRORP might as well be involved in the cleavage of non-tRNA transcripts. For instance, in Arabidopsis, published examples already show that PRORP1 is involved in the cleavage of the mitochondrial *nad6* and *orf291* RNAs at the level of predicted tRNA-like structures [22,67] and that PRORP2/3 are involved in the cleavage of a small nucleolar RNA (snoRNA) precursor in the nucleus [23]. Other challenges will be to understand the precise functions and mechanism for PRORP interactions with specific protein partners, i.e., TRMT10C and SDR5C1 in human mitochondria.

Answering the open questions regarding PRORP mode of action will rely on the determination of three-dimensional structures of PRORP either in complex with pre-tRNA substrates and/or with 5′ mature tRNA products. Such structures obtained either in solution or at atomic resolution will reveal the exact RNA binding and cleavage properties of PRORP and uncover the plasticity and dynamics of PRORP maturation of tRNA precursors.

## Figures and Tables

**Table 1:** Kinetic analyses of the importance of PRORP *cis*-elements for RNase P activity. At stands for *Arabidopsis thaliana*, Tt for *Thermus thermophilus*, “mito” for mitochondrial, “chloro” for chloroplastic, “nuc” for nuclear and “NA” for not applicable in the case of a leaderless RNA substrate. Grey boxes indicate results of single turnover kinetic experiments (with k_obs_, the observed first order constant obtained by the fit of a single exponential equation), whereas green boxes indicate results of RNase P cleavage assays performed in multiple turnover conditions (with k_cat_ and K_M_, the Michaelis-Menten parameters). K_D_ were derived from fluorescence polarization binding assays.

RNase P	Domain Mutated	Position Mutated	pre-tRNA	Cleavage (+/− or %)	k_obs_ (min^−1^)	k_cat_ (min^−1^)	K_M_ (nM)	K_D_ (nM)	References
AtPRORP1	-		At mito pre-tRNA^Cys^	+					[22]
	NYN	D474A/D475A		-					
	Nt deletion	Δ76			1.4 ± 0.1			700 ± 100	[19]
	Nt deletion	Δ245			<0.001			24,000 ± 10,000	
	NYN	D399A			<0.001			1100 ± 100	
	NYN	D474A			<0.001			1400 ± 600	
	NYN	D475A			<0.001			1400 ± 100	
	NYN	D493A			<0.001			300 ± 100	
	-				2.22 ± 0.12	3.72 ± 0.3	670 ± 230	510 ± 120	[57]
	-				1.2 ± 0.3			250 ± 34	[59]
	PPR 1	K101A			1.2 ± 0.4			859 ± 159	
	PPR 1	K109A			1.3 ± 0.3			389 ± 35	
	NYN	K439A			0.4 ± 0.1			609 ± 70	
	NYN	K460A			1.3 ± 0.1			377 ± 23	
	PPR 1	K101A/K109A			2.5 ± 0.1			1624 ± 344	
	PPR 1/NYN	K101A/K439A			0.5 ± 0.1			888 ± 127	
	PPR 1/NYN	K109A/K439A			0.5 ± 0.1			763 ± 92	
	-		Tt pre-tRNA^Gly^		2.3 ± 0.6				[60]
	-		At chloro pre-tRNA^Phe^	+					[22]
	NYN	D474A/D475A		-					
	-			100					[58]
	PPR 2	N136T		60					
	PPR 3	T180N		26					
	PPR 4	G215N		85					
	-			100					
	Nt deletion	Δ89		35					
	Domain deletion	ΔPPR1		3					
	Domain deletion	ΔPPR1-2		0					
	Domain deletion	ΔPPR1-3		0					
	Domain deletion	Δ89 PPR2		0					
	Domain deletion	Δ89 PPR3		0					[57]
	-				2.1 ± 0.12	2.52 ± 0.3	140 ± 50	60 ± 10	
	-		At nuc pre-tRNA^Cys^		2.22 ± 0.18	2.4 ± 0.12	550 ± 50	2300 ± 300	
	-		At nuc pre-tRNA^Phe^		4.68 ± 0.18	2.1 ± 0.18	160 ± 50	330 ± 60	
AtPRORP2	-		Tt pre-tRNA^Gly^		5.0 ± 1.2				[60]
	-		At nuc pre-tRNA^Gly^ 8:1		1.1 ± 0.1				[39]
	NYN	D393A			<0.001				
	NYN	D421A			<0.001				
	NYN	D422A			<0.001				
	NYN	D440A			<0.001				
	NYN	H445A			0.02 ± 0.004				
	Nt deletion	Δ141			<0.001				
	-		At mito pre-tRNA^Cys^		0.78 ± 0.18	0.78 ± 0.12	340 ± 60	350 ± 70	[57]
	-		At chloro tRNA^Phe^		1.08 ± 0.18	0.9 ± 0.12	340 ± 100	140 ± 10	
	-		At nuc pre-tRNA^Cys^		1.62 ± 0.12	1.8 ± 0.12	940 ± 130	6100 ± 2100	
	-		At nuc pre-tRNA^Phe^		2.1 ± 0.12	1.38 ± 0.12	250 ± 50	350 ± 40	
AtPRORP3	-		At pre-tRNA^Gln^				300 ± 90		[23]
	-		Tt pre-tRNA^Gly^		7.7 ± 2.7				[60]
	-		Tt pre-tRNA^Gly^		1.8 ± 0.1				[56]
	PPR 3	T113S			2.0 ± 0.1				
	PPR 3	R145N			2.0 ± 0.1				
	PPR 3	R145D			1.15 ± 0.02				
	PPR 3	T113N			1.56 ± 0.04				
	PPR 3	T113N-R145N			0.38 ± 0.02				
	PPR 3	T113N-R145D			0.047 ± 0.002				
	-		At mito pre-tRNA^Cys^		1.38 ± 0.12	1.32 ± 0.12	430 ± 30	300 ± 70	[57]
	-		At chloro pre-tRNA^Phe^		1.38 ± 0.12	0.78 ± 0.12	440 ± 50	220 ± 30	
	-		At nuc pre-tRNA^Cys^		1.80 ± 0.12	0.48 ± 0.12	420 ± 100	1500 ± 200	
	-		At nuc pre-tRNA^Phe^		4.32 ± 0.18	3.72 ± 1.38	2000 ± 850	380 ± 50	

**Table 2:** Kinetic analyses of the importance of pre-tRNA *cis*-elements for RNase P cleavage by PRORP enzymes. At stands for *Arabidopsis thaliana*, Tt for *Thermus thermophilus*, Bs for *Bacillus subtilis*, “mito” for mitochondrial and “nuc” for nuclear. L and T indicate lengths of leader and trailer sequences respectively. “Aa” substrates represent minimal substrates lacking the tRNA anticodon and D domains as described by Brillante et al. [56]. Values for the results published by Imai et al. [58] are graphical estimates as numbers were not provided in the article. Grey boxes indicate results of single turnover kinetic experiments (with either k_obs_, the observed first order constant, or k_react_, the maximum rate constant, and its associated K_M_), whereas green boxes indicate results of RNase P cleavage assays performed in multiple turnover conditions.

pre-tRNA	Type of Mutation on pre-tRNA	RNase P	% of Cleavage	k_obs_ or k_react_ (min^−1^)	K_M_ (nM)	K_D_ (nM)	References
At mito pre-tRNA^Cys^	-	AtPRORP1	100 ± 7				[20]
	ΔAC		75 ± 9				
	ΔDAC		0 ± 0				
	G18A		15 ± 2				
	G18C		10 ± 1				
	G19A		85 ± 3				
	G19C		90 ± 5				
	C56A		0 ± 0				
	C56G		0 ± 0				
	G57A		90 ± 6				
	G57C		10 ± 1				
	1CG72		100 ± 10				
	Δ3′		95 ± 1				
	3′ CCA		5 ± 2				
At chloro pre-tRNA^Phe^	-		100				[58]
	C56G		18				
	C56A		30				
	C56U		22				
	A57G		60				
	A57C		25				
	A57U		26				
	A58G		15				
	A58C		36				
	A58U		35				
	A59G		100				
	A59C		75				
	A59U		85				
At nuc pre-tRNA^Gly^	L23:T10	AtPRORP2		0.7 ± 0.1		118 ± 26	[39]
	L23:T05			1.0 ± 0.1		52 ± 12	
	L23:T01			0.7 ± 0.1		17 ± 5	
	L13:T01			0.7 ± 0.1		6 ± 1	
	L08:T01			1.1 ± 0.1		3 ± 1	
Tt pre-tRNA^Gly^	−(14)	AtPRORP3		1.67 ± 0.03	4.8 ± 0.4		[56]
	L7			1.7 ± 0.1	3.1 ± 0.7		
	L4			1.7 ± 0.1	3.4 ± 0.7		
	L2			1.6 ± 0.1	3.4 ± 0.8		
	L1			0.17 ± 0.02	5.4 ± 2.2		
	mature (CCA)			1.6 ± 0.1	4.9 ± 1.0		
	no trailer			1.5 ± 0.1	4.6 ± 0.7		
	40-nt trailer			1.5 ± 0.1	5.3 ± 1.1		
	-			1.67 ± 0.03	4.8 ± 0.4		
	U1-A72			2.2 ± 0.1	5.3 ± 0.9		
	U-1			2.9 ± 0.1	8.1 ± 1.4		
	G-1, A73			2.3 ± 0.1	6.5 ± 1.0		
	A-1, A73			5.1 ± 0.2	7.8 ± 1.4		
	A73			1.67 ± 0.04	4.5 ± 0.6		
	ΔAC			1.48 ± 0.04	1.7 ± 0.3		
	ΔD			0.36 ± 0.02	86 ± 16		
	AaT			0.066 ± 0.002	1839 ± 168		
	Aab1T			0.33 ± 0.01	1685 ± 218		
	Aab4T			0.26 ± 0.01	1151 ± 125		
	Aab9T			0.42 ± 0.01	40 ± 6		
	G18->A18			1.87 ± 0.07	22 ± 3		
	G19->A19/C56 > U56			1.78 ± 0.06	7.7 ± 1.2		
	C56->U56			1.81 ± 0.05	6.4 ± 0.9		
	A57->C57			1.56 ± 0.05	6.7 ± 0.9		
Bs pre-tRNA^Asp^	L0	AtPRORP1		NA		3400 ± 400	[57]
	L1			4.68 ± 0.18		150 ± 60	
	L2			9 ± 1.2		310 ± 20	
	L3			1.92 ± 0.06		140 ± 40	
	L4			1.5 ± 0.06		150 ± 40	
	L5			1.5 ± 0.06		190 ± 60	
	L10			1.5 ± 0.06		100 ± 50	
	L14			1.2 ± 0.06		100 ± 50	

## Abbreviations

The following abbreviations are used in this manuscript:

NYN	N4BP1, YacP-like Nuclease
PPR	pentatricopeptide repeat
P-RNA	ribonucleoprotein RNase P RNA subunit
Pre-tRNA	precursor tRNA
PRORP	protein-only RNase P
RNP	ribonucleoprotein
tRNA	transfer RNA
